# Survival outcomes of surgery and adjuvant chemotherapy in early-stage small cell and large cell lung cancer: a novel focus on tumors less than 1 cm

**DOI:** 10.1007/s12672-025-01777-z

**Published:** 2025-01-23

**Authors:** Jorge Raul Vazquez-Urrutia, Junjia Zhu, Shinkichi Takamori, Max Greenberg, Priyanka Bhatia, Takefumi Komiya

**Affiliations:** 1 Department of Medicine, Penn State Health Milton S. Hershey Medical Center, 500 University Dr, Hershey, PA 17033 USA; 2 Department of Public Health Sciences, Penn State College of Medicine, Hershey, PA 17033 USA; 3 Department of Thoracic and Breast Surgery, Oita University Faculty of Medicine, Oita, Japan; 4 Division of Hematology Oncology, Penn State College of Medicine, 500 University Dr, Hershey, PA 17033 USA

**Keywords:** Lung cancer, Small cell, Large cell, Early-stage, Adjuvant chemotherapy

## Abstract

**Background:**

The role of adjuvant chemotherapy in early-stage small cell lung cancer (SCLC) and large cell neuroendocrine carcinoma (LCNEC) remains unclear, particularly for small tumors. This study assesses the survival benefits of adjuvant chemotherapy after surgical resection with a novel focus on tumors less than 1 cm.

**Materials and methods:**

Data from the National Cancer Database (NCDB) was extracted for patients with SCLC (n = 11,962) and LCNEC (n = 6821) who underwent surgical resection between 2004 and 2020. Exclusion criteria were limited survival (< 30 days), positive lymph nodes, distant metastases, large tumors (> 5 cm), residual microscopic disease, and neoadjuvant therapy. The primary outcome was overall survival (OS) from diagnosis, which was evaluated using Kaplan–Meier methods and multivariate Cox regression analyses. A propensity score matching (PSM) analysis was performed to compare outcomes in patients with SCLC and tumors ≤ 1 cm who received adjuvant chemotherapy versus surgery alone.

**Results:**

The study involved 4114 SCLC and 3954 LCNEC patients. Adjuvant chemotherapy was associated with a significant increase median OS in both SCLC (6.26 vs. 4.18 years; p < 0.001) and LCNEC (7.02 years vs. 4.89 years; p < 0.001), while also being an independent predictor of better OS in SCLC (HR: 0.74) and LCNEC (HR: 0.75) (p < 0.001). The benefit was more noticeable in tumors ≤ 1 cm, showing a significant OS increase after PSM (median OS 7.34 vs. 5.02 years; p = 0.0048).

**Conclusion:**

Adjuvant chemotherapy after surgery is associated with improved overall survival in stage I SCLC and LCNEC, particularly in SCLC tumors of 1 cm or less.

**Supplementary Information:**

The online version contains supplementary material available at 10.1007/s12672-025-01777-z.

## Introduction

Lung cancer remains a burden for global medicine due to its high prevalence and lethality worldwide, being the leading cause of cancer mortality in males and the second in females. It is also more common in men than females. Only about 25.4% of individuals diagnosed with lung and bronchial cancers survive for 5 years or longer [1]. This statistic includes patients with both non–small cell lung cancer (NSCLC) and small cell lung cancer (SCLC) in the United States from 2013 to 2019 [1].

While there is no consensus on a staging system for SCLC, the 8th edition of American Joint Committee on Cancer (AJCC) classification categorizes patients with invasive tumors measuring 4 cm or smaller as stage I SCLC. Conversely, the VA system is broader, classifying any disease state from stage I to IIIB as limited stage (LS) and stage IV as extensive stage (ES) [2]. The American College of Chest Physicians and the European Society for Medical Oncology (ESMO) recommend that most cases be managed with a combination of radiation and chemotherapy for LS-SCLC. However, select patients with stage I SCLC may undergo surgical resection followed by adjuvant chemotherapy [2, 3]. The evidence supporting this recommendation is limited due to the lack of high-quality studies with most showing conflicting results [4, 5].

Similarly, for patients diagnosed with large cell neuroendocrine carcinoma of the lung (LCNEC), surgical resection plays a definitive role in treatment. However, the adjuvant chemotherapy regimens used for these individuals vary, and are based on NSCLC or SCLC treatment protocols. Consequently, the data supporting the efficiency of adjuvant chemotherapy in LCNEC is limited and inconsistent, leading to ongoing debate about its true benefit in this context [6].

Given the uncertainty and lack of evidence concerning the efficacy of adjuvant chemotherapy in early-stage SCLC and LCNEC, we conducted an analysis utilizing data from The National Cancer Database (NCDB), which encompasses information on approximately 70 percent of newly diagnosed cancer cases in the United States [7]. Our study aimed to compare the overall survival in those with small tumors, particularly 1 cm or less, between those receiving adjuvant chemotherapy and those who underwent surgical treatment alone.

## Material and methods

NCDB is a joint project of the Commission on Cancer (CoC) of the American College of Surgeons and the American Cancer Society. The CoC’s NCDB and the hospitals participating in the CoC NCDB are the sources of the deidentified data used herein; however, they have not verified and are not responsible for the statistical validity of the data analysis or the conclusions derived by the authors. The data are considered hospital-based rather than population- based [7]. Of note, this study was reviewed by the institutional review board at Penn State Health and was designated exempt from human subject research.

**Fig. 1:**
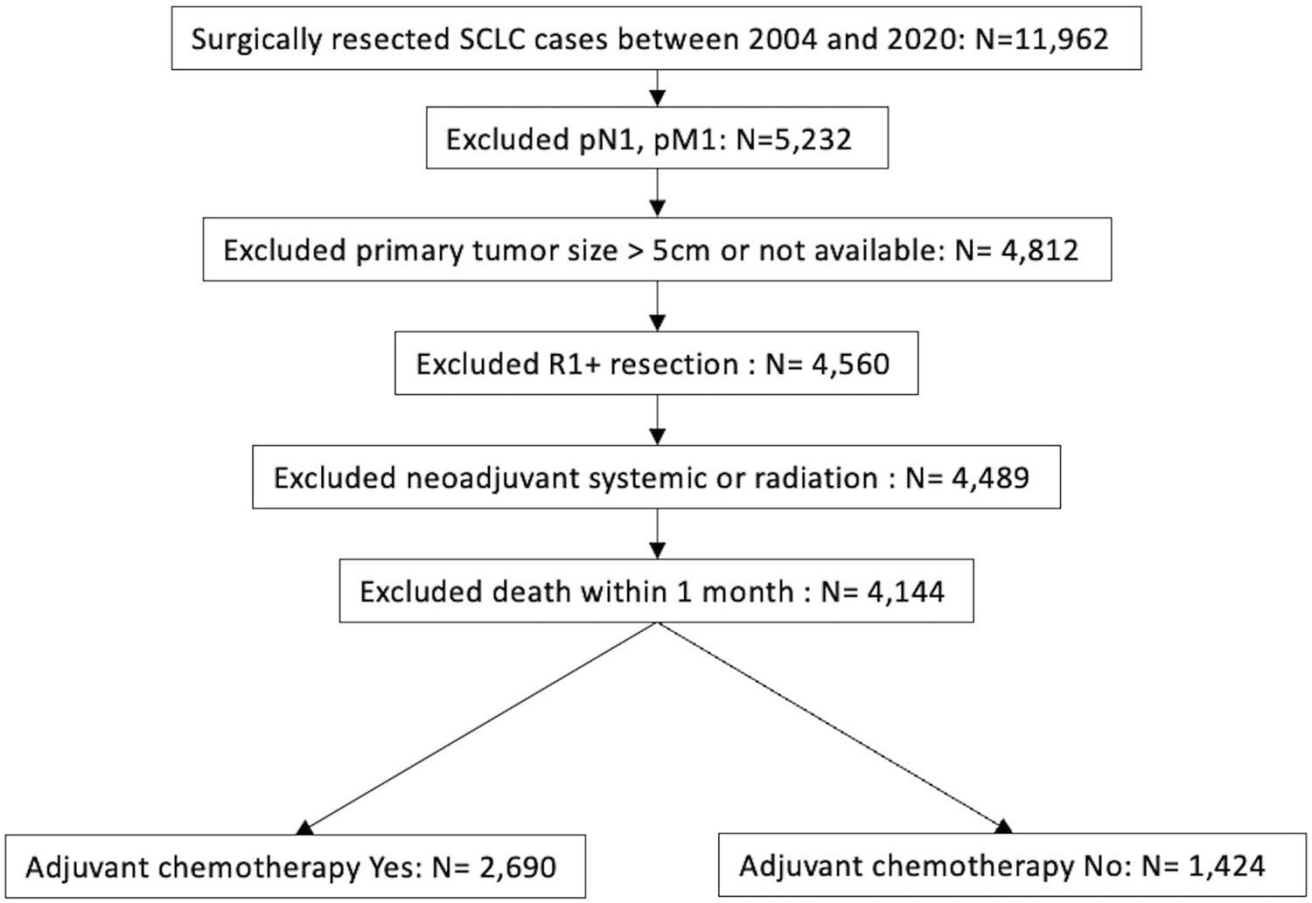
Selection criteria according to CONSORT diagram for small cell lung cancer cases. De-identified cases were released from National Cancer Database

Patients diagnosed with SCLC (n = 11,962) and LCNEC (n = 6,821) between 2004 and 2020 who underwent surgical resection were screened. Exclusions were made for those with positive lymph node, distant metastasis, tumors larger than 5 cm or with unspecified size, residual microscopic disease after resection, and those who had neoadjuvant chemotherapy and/or radiation. Additionally, patients who survived less than 30 days post-diagnosis were excluded (Fig. 1 and supplemental Fig. 1).

After applying initial inclusion and exclusion criteria, eligible patients were then assigned to a group of: (1) adjuvant chemotherapy in addition to surgery, (2) surgical resection alone.

The main outcome variable of this study is overall survival, which is defined as the length of time from the initial diagnosis to death of any causes or last follow-up. Key clinical characteristics were obtained and examined within each group. These included age (< 70 versus 70 or older), sex (male versus female), race (white versus others), institution (academic versus others), Charlson-Deyo comorbidity score (CD), year of diagnosis (2004–2015 versus 2016 +), Tumor size (≤ 1 cm versus 1 < T ≤ 3 cm versus 3 < T ≤ 5 cm), Lymphovascular invasion (LVI) (yes versus no), Visceropleuralinvasion (VPI) (yes versus no), and Adjuvant radiation (yes versus no). A propensity score matching analysis was conducted to compare groups (1) and (2) in SCLC with primary tumor size 1 cm or smaller.

## Statistical methods

The associations between clinical demographics and use of adjuvant chemotherapy were examined using Chi-square tests. Kaplan–Meier survival curves were generated and compared between treatment groups using log-rank tests. Univariate and multivariable Cox proportional hazard regression models were used to calculate hazard ratios (HRs) and their 95% confidence intervals (CIs) for survival since time of tissue diagnosis, and independent prognostic factors were identified. In the analysis of patients diagnosed with SCLC between 2004 and 2020 with primary tumor size less of 1 cm or less who underwent surgical resection, propensity score matching (PSM) was performed according to the XLSTAT software guideline. The PSM was performed using 1:1 nearest-neighbor matching and the following variables were used: age, sex, and surgery type. All other analyses were performed using JMP® 14.0 (SAS Institute Inc., Cary, NC, USA). All tests are two-sided and p < 0.05 was considered statistically significant.

## Results

Our sample selection process is detailed in Fig. 1 and supplemental Fig. 1. The study included 11,962 and 6821 patients diagnosed with SCLC and LCNEC between 2004 and 2020, respectively. After applying exclusion criteria, 4114 SCLC and 3954 LCNEC patients were assigned for group distribution. Among patients with SCLC, 2690 received adjuvant chemotherapy and 1424 underwent surgical resection alone. Similarly, 1081 and 2873 patients with LCNEC received adjuvant chemotherapy and underwent surgery alone, respectively.

The characteristics of patients and the associations between adjuvant chemotherapy use and clinical factors are detailed in Table 1. Patients with SCLC who received adjuvant chemotherapy were significantly younger than those who underwent surgery alone (< 70 years: 1652 [61%] vs. 672 [47%]; p < 0.001), more likely to be diagnosed after 2016 (1154 [43%] vs 530 [37%]; p < 0.001), and more likely to receive adjuvant radiation (767 [29%] vs. 38 [3%]; p < 0.001). Similarly, LCNEC patients who received adjuvant chemotherapy were younger than those managed surgically (< 70 years: 752 [70%] vs. 1577 [55%]; p < 0.001), had larger overall tumor sizes (3 < T ≤ 5 cm: 387 [36%] vs. 582 [21%]; p < 0.001), and were more likely to receive adjuvant radiation (114[11%] vs. 57[2%]; p < 0.001). Notably, these patients were also more likely to exhibit LVI(201 [19%] vs. 443 [15%]; p < 0.05) and VPI (124 [11%] vs. 230 [8%]; p < 0.001).For SCLC patients with primary tumor size ≤ 1 cm, we successfully matched 151 patients who underwent surgery alone with 151 patients who also received adjuvant chemotherapy, using propensity score matching method. The characteristics after PSM are shown in supplemental Table 1. After PSM, groups were well balanced in characteristics except for the year of diagnosis and adjuvant radiation receiving status.

**Table 1:** Clinical characteristics of patients with pT1-2N0 SCLC and LCNEC with or without adjuvant chemotherapy

Factors	SCLC (n = 4114)			LCNEC (n = 3954)		
	Adjuvant chemotherapy			Adjuvant chemotherapy		
	Yes (n = 2690)	No (n = 1424)	p-value	Yes (n = 1081)	No (n = 2873)	p-value
Institution						
Academic	921 (34%)	535 (38%)	0.0335	336 (31%)	1088 (38%)	< 0.0001
Other	1769 (66%)	889 (62%)		745 (69%)	1785 (62%)	
Age						
≥ 70	1038 (39%)	752 (53%)	< 0.0001	329 (30%)	1296 (45%)	< 0.0001
< 70	1652 (61%)	672 (47%)		752 (70%)	1577 (55%)	
Sex						
Male	1224 (46%)	638 (45%)	0.6685	514 (48%)	1422 (49%)	0.2751
Female	1466 (54%)	786 (55%)		567 (52%)	1451 (51%)	
Race						
White	2473 (92%)	1283 (90%)	0.0470	969 (90%)	2518 (88%)	0.0831
Other	217 (8%)	141 (10%)		112 (10%)	355 (12%)	
CD score						
0–1	2147 (80%)	1146 (80%)	0.6125	879 (81%)	2274 (79%)	0.1315
2–3	543 (20%)	278 (20%)		202 (19%)	599 (21%)	
Year of diagnosis						
2004–2015	1536 (57%)	894 (63%)	0.0004	681 (63%)	1892 (66%)	0.0930
2016 +	1154 (43%)	530 (37%)		400 (37%)	981 (34%)	
Tumor size						
≤ 1cm	267 (10%)	151 (11%)	0.7908	55 (5%)	239 (8%)	< 0.0001
1 < T ≤ 3cm	462 (17%)	243 (17%)		639 (59%)	2052 (71%)	
3 < T ≤ 5 cm	1961 (73%)	1030 (72%)		387 (36%)	582 (21%)	
Surgery type						
Lobectomy +	1916 (71%)	1049 (74%)	0.0972	812 (75%)	2055 (72%)	0.0243
Sublobar	774 (29%)	375 (26%)		269 (25%)	818 (28%)	
Adjuvant Radiation						
Yes	767 (29%)	38 (3%)	< 0.0001	114 (11%)	57 (2%)	< 0.0001
No	1923 (71%)	1386 (97%)		967 (89%)	2816 (98%)	
LVI						
Yes	551 (20%)	257 (18%)	0.0614	201 (19%)	443 (15%)	0.0160
No	2139 (80%)	1167 (82%)		880 (81%)	2430 (85%)	
VPI						
Yes	231 (9%)	112 (8%)	0.4254	124 (11%)	230 (8%)	0.0007
No	2459 (91%)	1312 (92%)		957 (89%)	2643 (92%)	

*CD* Charlson-Deyo, *Adeno* adenocarcinoma, *LVI* lymphovascular invasion, *VPI* visceral pleural invasion

### *Adjuvant chemotherapy is associated with improved overall survival in SCLC and LCNEC patients with tumors* ≤ *5 cm*

**Fig. 2:**
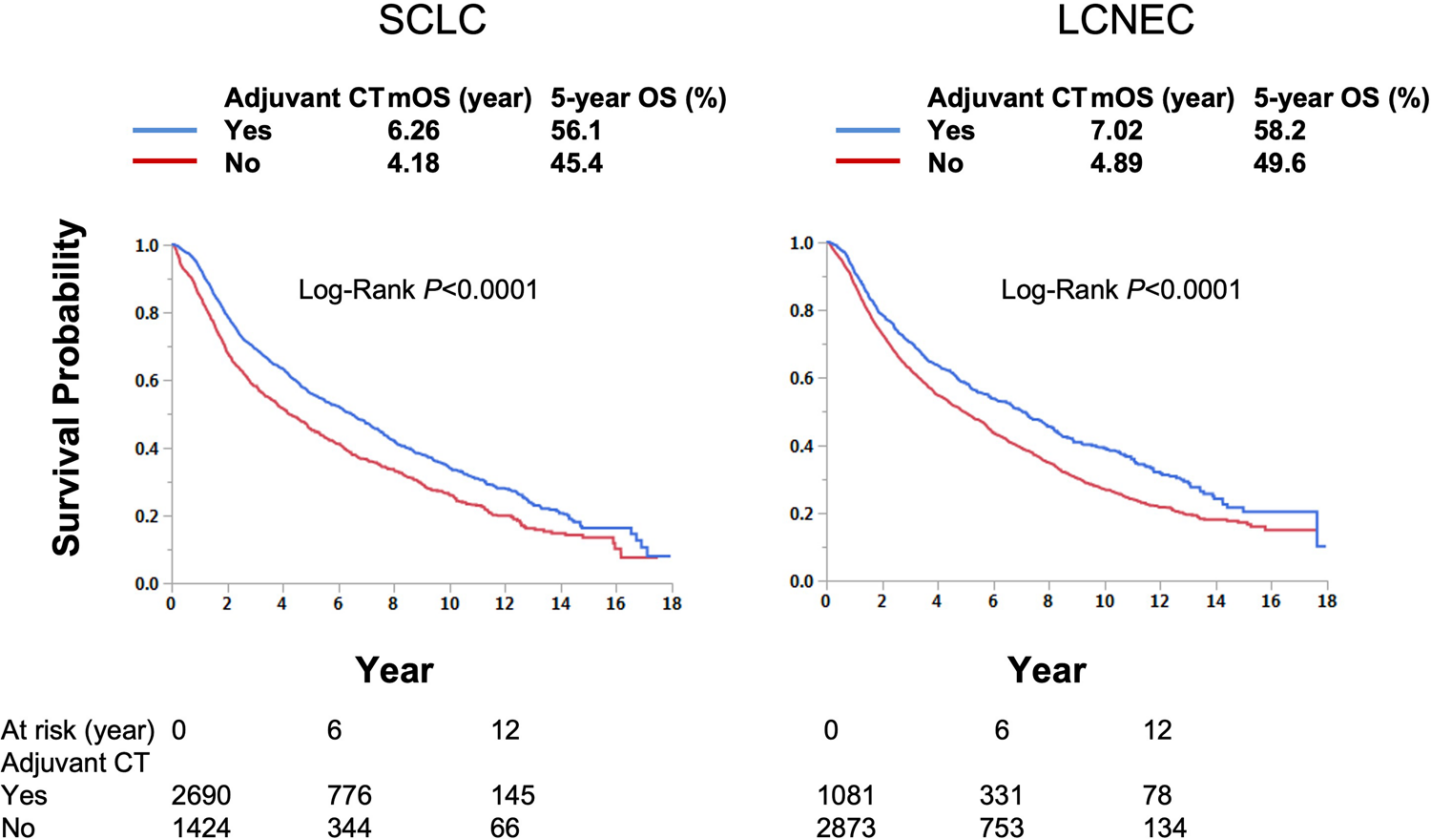
Adjuvant chemotherapy improves overall survival in patients with early-stage SCLC and LCNEC, compared to surgery alone. Median survival years and log-rank P-values are presented. *mOS* median overall survival, *SCLC* small cell lung cancer, *LCNEC* large cell neuroendocrine carcinoma

Kaplan Meir analysis demonstrated that, compared to surgery alone, adjuvant chemotherapy significantly prolonged median OS and 5-year OS rate in patients with pT1-2 N0 SCLC (mOS: 6.26 year vs 4.18 year p < 0.001; 5-year OS 56.1% vs 45.4%) and LCNEC (mOS: 7.02 year vs 4.89 year p < 0.001; 5-year OS 58.2% vs 49.6%) (Fig. 2). Adjuvant chemotherapy was proved to be an independent predictor of improved OS in univariate and multivariate analysis in both the SCLC group (HR: 0.73 and 0.74; both p < 0.001, respectively) and LCNEC group (HR: 0.76 and 0.75; both p < 0001, respectively) (Table 2). Other variables associated with improved OS were age younger than 70, female sex, lower CD score, diagnosis on and after 2016, smaller tumor size, lobectomy as type of surgery as well as absence of LVI and VPI (Table 2) for both SCLC and LCNEC in uni- and multivariate analyses, and non-white race, and use of adjuvant radiation for LCNEC in uni- and multivariate analyses.

**Table 2:** Univariate and multivariable Cox regression analyses for overall survival in pT1-2N0 SCLC and LCNEC patients with tumor size ≤ 5 cm

Factors		SCLC		LCNEC	
		Univariate	Multivariable	Univariate	Multivariable
		HR (95% CI)	HR (95% CI)	HR (95% CI)	HR (95% CI)
		p value	p value	p value	p value
Institution	Academic/others (Ref)	0.99 (0.91–1.08)	0.95 (0.87–1.03)	0.88 (0.80–0.96)	0.89 (0.82–0.97)
		p = 0.7991	p = 0.2134	p = 0.0027	p = 0.0100
Age	< 70/ 70 ≤ (Ref)	0.62 (0.57–0.68)	0.67 (0.62–0.73)	0.56 (0.51–0.60)	0.60 (0.55–0.65)
		p < 0.0001	p < 0.0001	p < 0.0001	p < 0.0001
Sex	Female/male (Ref)	0.83 (0.76–0.90)	0.84 (0.77–0.91)	0.84 (0.77–0.91)	0.86 (0.79–0.94)
		p < 0.0001	p < 0.0001	p < 0.0001	p = 0.0005
Race	Others/white (Ref)	0.95 (0.81–1.10)	0.96 (0.83–1.12)	0.74 (0.65–0.85)	0.80 (0.70–0.92)
		p = 0.5018	p = 0.6455	p < 0.0001	p = 0.0019
CD Score	0–1/2 ≤ (Ref)	0.81 (0.73–0.90)	0.83 (0.75–0.92)	0.78 (0.70–0.86)	0.79 (0.72–0.88)
		p < 0.0001	p = 0.0004	p < 0.0001	p < 0.0001
Year of Diagnosis	2016 + /2004–2015 (Ref)	0.89 (0.80–0.98)	0.86 (0.78–0.95)	0.73 (0.66–0.82)	0.72 (0.65–0.81)
		p = 0.0146	p = 0.0028	p < 0.0001	p < 0.0001
Tumor size	≤ 1 cm/3–5 cm (Ref)	0.77 (0.65–0.91)	0.73 (0.62–0.87)	0.70 (0.58–0.84)	0.60 (0.49–0.72)
		p = 0.0016	p = 0.0003	p < 0.0001	p < 0.0001
	1 < T ≤ 3 cm/3–5 cm (Ref)	0.93 (0.83–1.03)	0.89 (0.80–0.99)	0.90 (0.82–0.99)	0.78 (0.71–0.86)
		p = 0.1715	p = 0.0390	p = 0.0263	p < 0.0001
Surgery	Lobectomy + /sub(Ref)	0.68 (0.62–0.74)	0.69 (0.63–0.75)	0.67 (0.61–0.73)	0.69 (0.63–0.75)
		p < 0.0001	p < 0.0001	p < 0.0001	p < 0.0001
Adjuvant Radiation	Yes/no (Ref)	0.94 (0.85–1.04)	1.11 (1.00–1.24)	1.33 (1.11–1.58)	1.45 (1.20–1.73)
		p = 0.2388	p = 0.0509	p = 0.0026	p < 0.0001
LVI	No/yes (Ref)	0.82 (0.74–0.91)	0.81 (0.73–0.90)	0.81 (0.73–0.91)	0.77 (0.68–0.86)
		p = 0.0003	p = 0.0002	p = 0.0006	p < 0.0001
VPI	No/yes (Ref)	0.76 (0.66–0.87)	0.82 (0.72–0.95)	0.95 (0.82–1.10)	0.98 (0.84–1.13)
		p = 0.0001	p = 0.0067	p = 0.8249	p = 0.7584
Adjuvant chemotherapy	Yes/no (Ref)	0.73 (0.67–0.79)	0.74 (0.67–0.81)	0.76 (0.69–0.83)	0.75 (0.67–0.82)
		p < 0.0001	p < 0.0001	p < 0.0001	p < 0.0001

*SCLC* small cell lung cancer, *LCNEC* large cell neuroendocrine carcinoma, *HR* hazard ratio, *CI* confidence interval, *Ref* reference, *CD* Charlson Comorbidity, *Sub* sublobar, *LVI* lymphovascular invasion, *VPI* visceral pleural invasion

### *Adjuvant chemotherapy benefit in overall survival is more pronounced in SCLC with tumors* ≤ *1 cm*

**Fig. 3:**
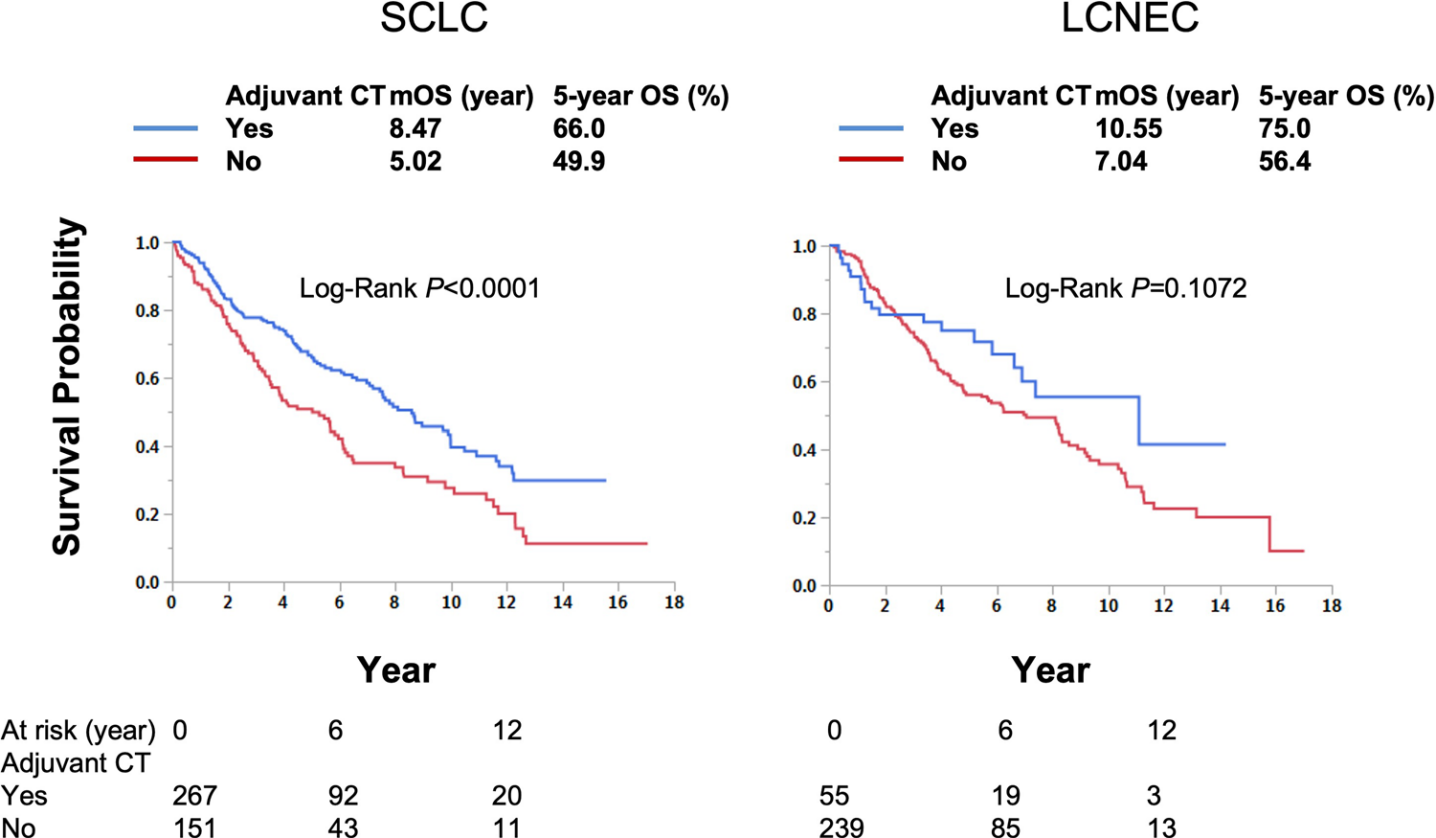
Adjuvant chemotherapy improves overall survival in SCLC and LCNEC patients with tumors ≤ 1 cm compared to surgery alone. Median survival years and log-rank P-values are presented. *mOS* median overall survival, *SCLC* small cell lung cancer, *LCNEC* large cell neuroendocrine carcinoma

We specifically evaluated the effect in median and 5-year overall survival in those with tumor size of ≤ 1 cm. Notably, this effect was more pronounced in SCLC (mOS: 8.47 year vs 5.02 year p < 0.001; 5-year OS 66% vs 49.9%), not in LCNEC (mOS: 10.55 year vs 7.04 year, p = 0.1072; 5-year OS 75% vs 56.4%) patient groups (Fig. 3). We confirmed the effect of this intervention through PSM in those the SCLC patient subgroup, which showed a statistically significant increase compared to the surgery alone group (mOS: 7.34 year vs 5.02 year; 5 year-OS 61.5% and 49.9%, p = 0.0048) (Supplemental Fig. 2) and was shown to be an independent prognostic factor in these patients (HR: 0.62; p = 0.0152) (Table 3). Interestingly, lobectomy also showed to be associated with improved OS, compared to sub lobar lung resection.

**Table 3:** Univariate and multivariable Cox regression analyses for overall survival in SCLC patients with tumor size ≤ 1 cm: pre and post propensity-score matching analysis to compare by adjuvant chemotherapy status

Factors	N (Adjuvant chemotherapy Yes/No)	Pre-PSM		Post-PSM	
		267/151		151/151	
		Univariate	Multivariable	Univariate	Multivariable
		HR (95% CI)	HR (95% CI)	HR (95% CI)	HR (95% CI)
		P value	P value	P value	P value
Institution	Academic/others (Ref)	1.24 (0.93–1.64)	1.17 (0.88–1.56)	1.14 (0.81–1.60)	1.06 (0.74–1.49)
		p = 0.1391	p = 0.2767	p = 0.4324	p = 0.7533
Age	< 70/ 70 ≤ (Ref)	0.58 (0.44–0.76)	0.64 (0.49–0.85)	0.60 (0.43–0.82)	0.62 (0.44–0.87)
		p < 0.0001	p = 0.0018	p = 0.0017	p = 0.0056
Sex	Female/male (Ref)	0.72 (0.55–0.96)	0.74 (0.56–0.98)	0.85 (0.62–1.17)	0.74 (0.54–1.04)
		p = 0.0228	p = 0.0350	p = 0.3234	p = 0.0792
Race	Others/white (Ref)	0.90 (0.52–1.45)	0.92 (0.52–1.50)	1.05 (0.59–1.73)	1.12 (0.62–1.88)
		p = 0.6829	p = 0.7440	p = 0.8657	p = 0.6883
CD Score	0–1/2 ≤ (Ref)	0.79 (0.57–1.12)	0.79 (0.56–1.13)	0.68 (0.46–1.04)	0.74 (0.49–1.14)
		p = 0.1784	p = 0.1877	p = 0.0759	p = 0.1653
Year of Diagnosis	2016 + /2004–2015 (Ref)	0.79 (0.55–1.11)	0.81 (0.56–1.16)	0.68 (0.47–0.98)	0.81 (0.54–1.21)
		p = 0.1785	p = 0.2520	p = 0.0395	p = 0.3105
Surgery	Lobectomy + /sub(Ref)	0.60 (0.46–0.79)	0.63 (0.47–0.83)	0.64 (0.46–0.88)	0.66 (0.48–0.93)
		p = 0.0003	p = 0.0012	p = 0.0066	p = 0.0163
Adjuvant Radiation	Yes/no (Ref)	0.93 (0.66–1.29)	1.13 (0.78–1.62)	0.96 (0.59–1.47)	1.21 (0.72–1.97)
		p = 0.6860	p = 0.5076	p = 0.8558	p = 0.4550
LVI	No/yes (Ref)	0.68 (0.45–1.07)	0.68 (0.44–1.09)	0.66 (0.42–1.09)	0.68 (0.41–1.17)
		p = 0.0927	p = 0.1048	p = 0.0975	p = 0.1594
VPI	No/yes (Ref)	1.34 (0.68–3.14)	1.57 (0.77–3.78)	1.14 (0.52–3.23)	1.25 (0.54–3.63)
		p = 0.4312	p = 0.2270	p = 0.7621	p = 0.6301
Adjuvant chemotherapy	Yes/no (Ref)	0.59 (0.45–0.77)	0.63 (0.47–0.85)	0.62 (0.44–0.86)	0.62 (0.42–0.91)
		p = 0.0002	p = 0.0022	p = 0.0044	p = 0.0152

*SCLC* small cell lung cancer, *HR* hazard ratio, *CI* confidence interval, *Ref* reference, *CD* Charlson Comorbidity, *Sub* sublobar, *LVI* lymphovascular invasion, *VPI* visceral pleural invasion

## Discussion

SCLC often presents at an advance stage with early metastasis. As such, current treatment guidelines advocate for multimodal chemotherapy and radiotherapy. This constitutes the rationale for some guidelines to conceptualize management between LS and ESgiven the aggressive nature of this disease and rarity of early-stage diagnosis. Historically, the National Comprehensive Cancer Network and ESMO acknowledge that a subset of patients with stage I disease may benefit from surgical resection followed by adjuvant chemotherapy [2, 3, 8]. This is based on retrospective studies and small prospective trials indicating that adjuvant chemotherapy can reduce recurrence rates and potentially improve OS compared to surgery alone [5, 9–11].

In this context, a Cochrane systematic review by Barnes et al. pooled data from several randomized studies from 1946 to 2017 found a lack of high-quality and contemporary evidence to evaluate the role of surgery with adjuvant chemotherapy for early SCLC, as these studies were employed by outdated staging systems and therapeutic regimens [5]. Another meta-analysis by Liu et al. analyzed 2 randomized control trials (RCTs) and 13 retrospective studies. While retrospective studies indicated that surgery-based multimodal therapy improved OS for stage I disease, the RCTs did not demonstrate this benefit [9]. Moreover, Martucci et al. described 7 prospective studies showing that this approach is feasible and can achieve a 5-year survival rate of 36–63% for patients with completely resected stage I SCLC. These findings were supported by 9 retrospective and large population-based studies over the past 40 years [10]. Additionally, a study Yang et al. extracted data from the NCDB database and found that adjuvant therapy, including chemotherapy alone or with radiation, significantly improved survival compared to surgery alone [11]. Overall, this points towards a potentially beneficial role of adjuvant chemotherapy to surgical treatment in early-stage SCLC. However, a fundamental limitation is the reliance on small sample studies and the absence of recent studies, largely due to the discouragement generated by the available clinical trials.

Additionally, LCNEC is traditionally treated similarly to NSCLC in early stages, with surgery being preferred for stage I disease. However, given its neuroendocrine features, there has been growing interest in the potential role of adjuvant chemotherapy. In line with this, a meta-analysis by Chen et al. analyzed 15 retrospective studies, which demonstrated an overall increase in OS in stages I-III compared to surgical management alone [12]. Another study by Shen et al. found that adjuvant chemotherapy, particularly etoposide-platinum based regimens were associated with improved OS in LCNEC patients [13]. An earlier study by Sarkaria et al. echoed this finding in a retrospective cohort study on patients with stage I LCNEC receiving adjuvant platinum-based chemotherapy after surgery [14].

Our study analyzed utilized data from the NCDB, the largest clinical cancer database to date and demonstrated that in stage I SCLC and LCNEC, adjuvant chemotherapy use after surgical resection of the primary tumor was associated with increased median and 5 year-OS in both univariate and multivariate analysis. Unlike other studies including Yang et al., our study is the first to our knowledge to evaluate outcomes associated with tumor size, particularly tumors ≤ 1 cm. The improvement in OS was more noticeable in the subset of patients with tumors ≤ 1 cm, which could be explained by the early dissemination and chemo-sensitiveness of SCLC and LCNEC. Furthermore, this effect was preserved in SCLC after PSM analysis, decreasing the likelihood of bias in our results.

Another relevant finding in our study involved the type of surgery used to treat small tumors. Recent pivotal studies have confirmed that sub lobar resection is non-inferior to lobectomy in patients diagnosed with peripheral NSCLC with tumors up to 2 cm in size [15, 16]. Notably, in patients with sub centimeter NSCLC, retrospective studies have suggested that segmentectomy offers oncologic outcomes comparable to lobectomy [17, 18]. However, our multivariate analysis of OS demonstrated that lobectomy was associated with significantly longer OS than sub lobar resection. Therefore, in patients with sub centimeter SCLC, lobectomy may still be necessary, even for small tumors.

It is certainly important to acknowledge some of the limitations inherent in our study. Firstly, the non-randomized assignment of patients to treatment groups and the retrospective nature of our analysis introduces inherent biases. To mitigate these, PSM and multivariate analysis were employed.

Furthermore, while the NCDB serves as a valuable resource for cancer related research, it does not include certain relevant clinical data, such as ECOG performance status, disease-free survival, chemotherapy regimen, number of treatment cycles, treatment after disease progression, details on treatment-related adverse events, and concurrent non-cancer medications. This is of relevance in SCLC and LCNEC because of the known development of chemoresistance in these tumors.

Additionally, our findings lack external validation from independent cohorts, demonstrating the need for further validation to strengthen the robustness of our results. Moving forward, prospective studies incorporating a more comprehensive array of variables are warranted to enhance the evidential support for our findings.

In conclusion, this retrospective study, utilizing the largest cancer database, suggests that adjuvant chemotherapy could be considered a standard component of treatment for patients with stage I SCLC and LCNEC following surgical resection as it is associated with favorable clinical outcomes. Notably, our study is unique in evaluating the impact of adjuvant chemotherapy on tumors 1 cm or less in size, a finding that has not been reported elsewhere.

## Supplementary Information

Supplementary material 1: Figure 1: Selection criteria according to CONSORT diagram for LCNEC cases. De-identified cases were released from the National Cancer Database. *LCNEC* large cell neuroendocrine carcinoma. Figure 2: Adjuvant chemotherapy improves overall survival in SCLC patients with tumors ≤1 cm compared to surgery alone, as demonstrated by PSM analysis. Median survival years and log-rank P-values are reported, with matched cases compared for overall survival. *PSM* propensity score matching, *SCLC* small cell lung cancer

## Data Availability

The datasets analyzed during the current study are available via NCDB upon request.
