# Supplementary material for: Survival outcomes of surgery and adjuvant chemotherapy in early-stage small cell and large cell lung cancer: a novel focus on tumors less than 1 cm
Source: Discov Oncol. 2025 Jan 23;16:82. doi: 10.1007/s12672-025-01777-z (PMC11757834; doi:10.1007/s12672-025-01777-z)
Supplement: Supplementary file 1 — Supplementary material 1: Figure 1: Selection criteria according to CONSORT diagram for LCNEC cases. De-identified cases were released from the National Cancer Database. LCNEC large cell neuroendocrine carcinoma. Figure 2: Adjuvant chemotherapy improves overall survival in SCLC patients with tumors ≤1 cm compared to surgery alone, as demonstrated by PSM analysis. Median survival years and log-rank P-values are reported, with matched cases compared for overall survival. PSM propensity score matching, SCLC small cell lung cancer [file 12672_2025_1777_MOESM1_ESM.zip › 12672_2025_1777_MOESM1_ESM/New folder/Supplemental Table 1. PSM.docx]

Supplemental Table 1: Clinical characteristics of SCLC patients with tumor size ≤ 1cm: pre and post propensity-score matching

Factors Pre-PSM Post-PSM

Adjuvant chemotherapy Adjuvant chemotherapy

Yes (n=267) No (n=151) P-value Yes (n=151) No (n=151) P-value

Institution

Academic 95 (36%) 53 (35%) 0.9213 56 (37%) 53 (35%) 0.7193

Other 172 (64%) 98 (65%) 95 (63%) 98 (65%)

Age

≥ 70 110 (41%) 74 (49%) 0.1224 74 (49%) 74 (49%) 1.0000

< 70 157 (59%) 77 (51%) 77 (51%) 77 (51%)

Sex

Male 90 (34%) 62 (41%) 0.1334 62 (41%) 62 (41%) 1.0000

Female 177 (66%) 89 (59%) 89 (59%) 89 (59%)

Race

White 249 (93%) 136 (90%) 0.2450 138 (91%) 136 (90%) 0.6915

Other 18 (7%) 15 (10%) 13 (9%) 15 (10%)

CD score

0-1 219 (82%) 124 (82%) 0.9802 126 (83%) 124 (82%) 0.7605

2-3 48 (18%) 27 (18%) 25 (17%) 27 (18%)

Year of diagnosis

2004-2015 156 (58%) 104 (69%) 0.0343 47 (31%) 104 (69%) <0.0001

2016+ 111 (42%) 47 (31%) 104 (69%) 47 (31%)

Surgery type

Lobectomy+ 161 (60%) 91 (60%) 0.9944 91 (60%) 91 (60%) 1.0000

Sublobar 106 (40%) 60 (40%) 60 (40%) 60 (40%)

Adjuvant Radiation

Yes X X <0.0001 X X <0.0001

No Y Y Y Y

LVI

Yes 28 (10%) 19 (13%) 0.5147 19 (13%) 19 (13%) 1.0000

No 239 (90%) 132 (87%) 132 (87%) 132 (87%)

VPI

Yes X X 0.2443 X X 0.3100

No Y Y Y Y

SCLC, small cell lung cancer; PSM, propensity-score matching; CD, Charlson-Deyo; LVI, lymphovascular invasion; VPI, visceral pleural invasion; X and Y number less than 10 cannot be reported according to NCDB agreement
