# Supplementary figures and images for: Survival outcomes of surgery and adjuvant chemotherapy in early-stage small cell and large cell lung cancer: a novel focus on tumors less than 1 cm
Source: Discov Oncol. 2025 Jan 23;16:82. doi: 10.1007/s12672-025-01777-z (PMC11757834; doi:10.1007/s12672-025-01777-z)

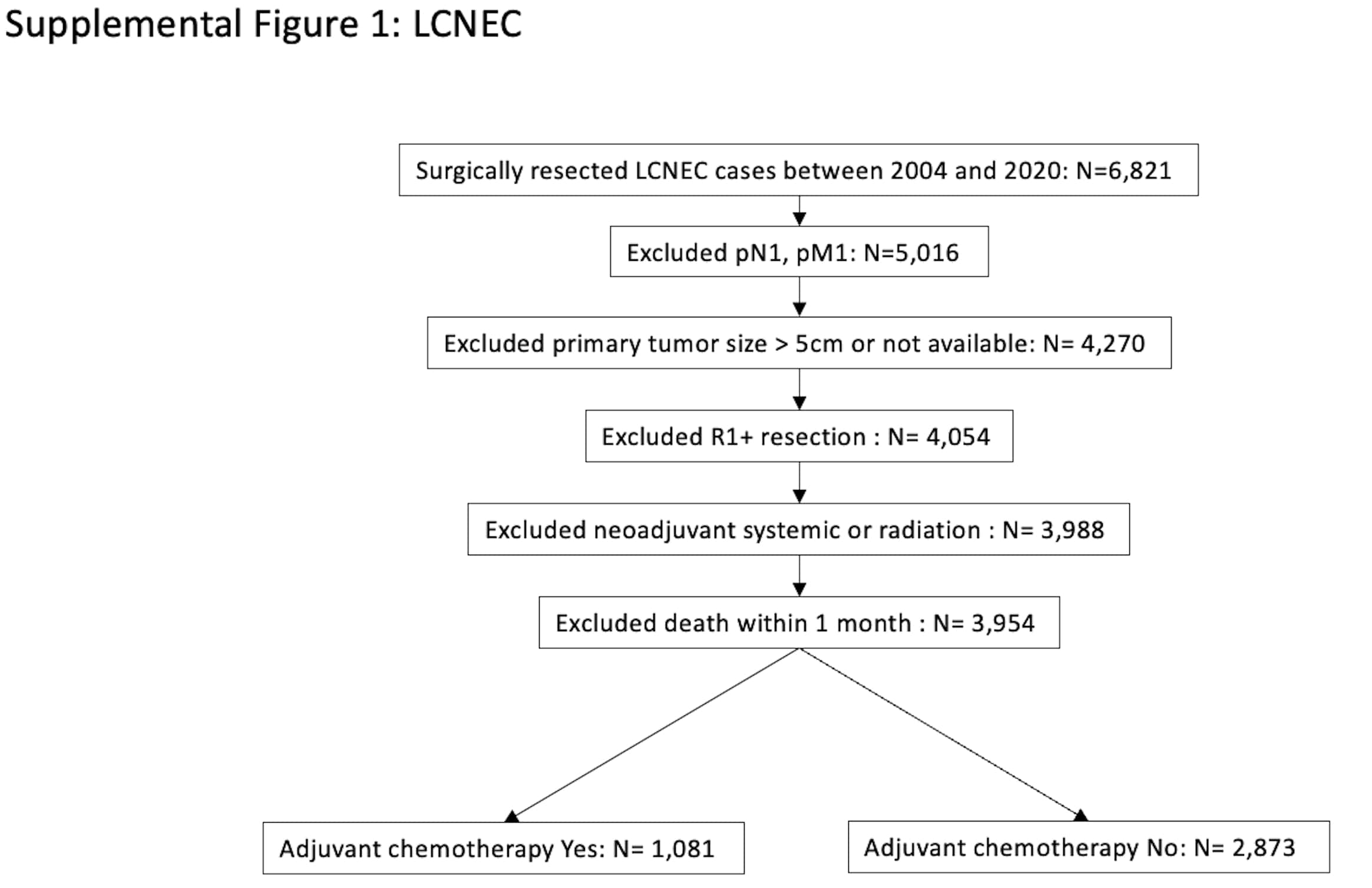

Supplement: Supplementary file 1 — Supplementary material 1: Figure 1: Selection criteria according to CONSORT diagram for LCNEC cases. De-identified cases were released from the National Cancer Database. LCNEC large cell neuroendocrine carcinoma. Figure 2: Adjuvant chemotherapy improves overall survival in SCLC patients with tumors ≤1 cm compared to surgery alone, as demonstrated by PSM analysis. Median survival years and log-rank P-values are reported, with matched cases compared for overall survival. PSM propensity score matching, SCLC small cell lung cancer [file 12672_2025_1777_MOESM1_ESM.zip › 12672_2025_1777_MOESM1_ESM/New folder/Supp Fig1.jpg]

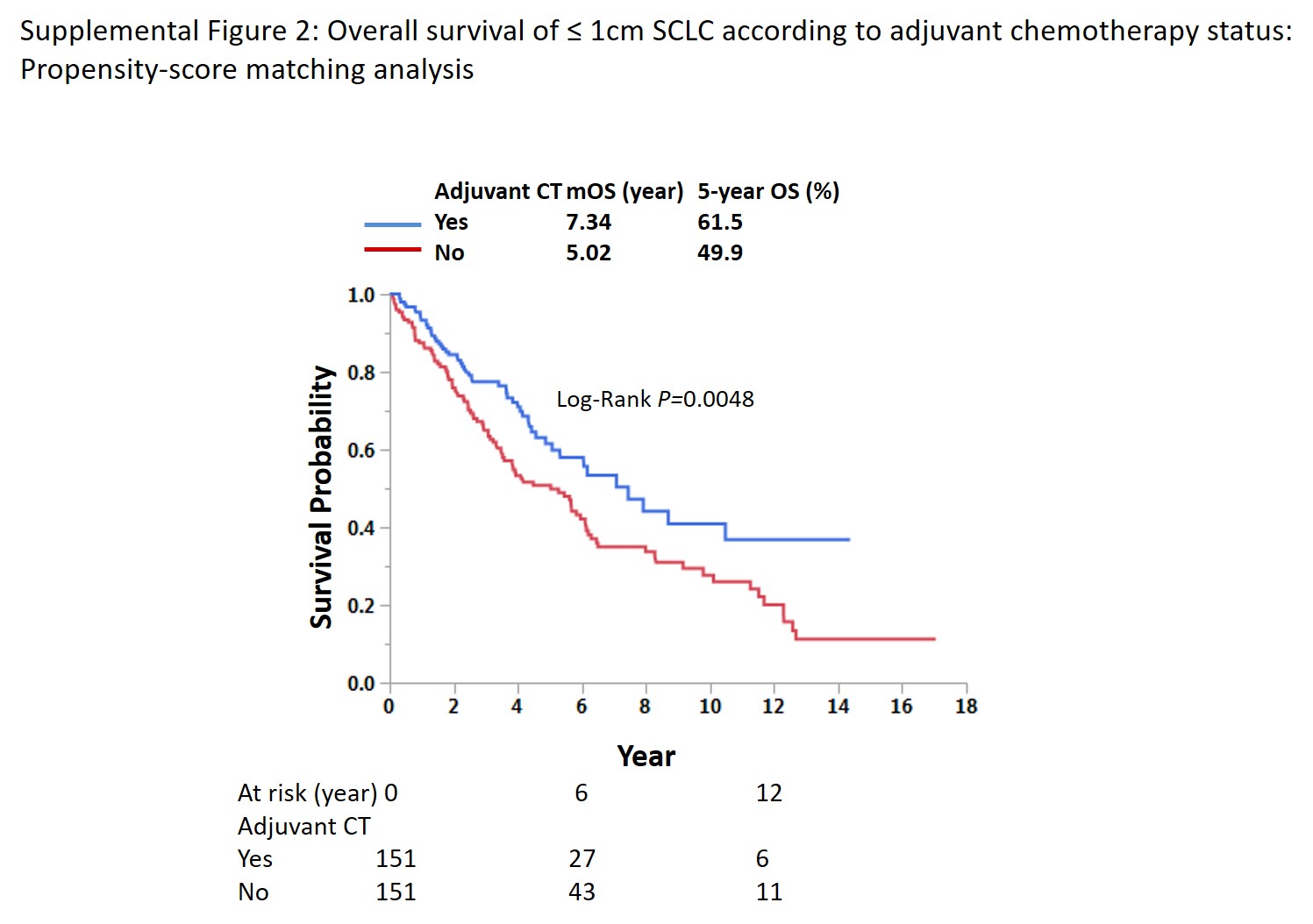

Supplement: Supplementary file 1 — Supplementary material 1: Figure 1: Selection criteria according to CONSORT diagram for LCNEC cases. De-identified cases were released from the National Cancer Database. LCNEC large cell neuroendocrine carcinoma. Figure 2: Adjuvant chemotherapy improves overall survival in SCLC patients with tumors ≤1 cm compared to surgery alone, as demonstrated by PSM analysis. Median survival years and log-rank P-values are reported, with matched cases compared for overall survival. PSM propensity score matching, SCLC small cell lung cancer [file 12672_2025_1777_MOESM1_ESM.zip › 12672_2025_1777_MOESM1_ESM/New folder/Supplemental figure 2-SCLC-corrected.jpg]
